# Gender Disparity in the Authorship of Biomedical Research Publications During the COVID-19 Pandemic: Retrospective Observational Study

**DOI:** 10.2196/25379

**Published:** 2021-04-12

**Authors:** Goran Muric, Kristina Lerman, Emilio Ferrara

**Affiliations:** 1 Information Sciences Institute University of Southern California Los Angeles, CA United States; 2 Department of Computer Science University of Southern California Los Angeles, CA United States; 3 Annenberg School for Communication and Journalism University of Southern California Los Angeles, CA United States

**Keywords:** science of science, gender disparities, research evaluation, COVID-19

## Abstract

**Background:**

Gender imbalances in academia have been evident historically and persist today. For the past 60 years, we have witnessed the increase of participation of women in biomedical disciplines, showing that the gender gap is shrinking. However, preliminary evidence suggests that women, including female researchers, are disproportionately affected by the COVID-19 pandemic in terms of unequal distribution of childcare, elderly care, and other kinds of domestic and emotional labor. Sudden lockdowns and abrupt shifts in daily routines have had disproportionate consequences on their productivity, which is reflected by a sudden drop in research output in biomedical research, consequently affecting the number of female authors of scientific publications.

**Objective:**

The objective of this study is to test the hypothesis that the COVID-19 pandemic has had a disproportionate adverse effect on the productivity of female researchers in the biomedical field in terms of authorship of scientific publications.

**Methods:**

This is a retrospective observational bibliometric study. We investigated the proportion of male and female researchers who published scientific papers during the COVID-19 pandemic, using bibliometric data from biomedical preprint servers and selected Springer-Nature journals. We used the ordinary least squares regression model to estimate the expected proportions over time by correcting for temporal trends. We also used a set of statistical methods, such as the Kolmogorov-Smirnov test and regression discontinuity design, to test the validity of the results.

**Results:**

A total of 78,950 papers from the bioRxiv and medRxiv repositories and from 62 selected Springer-Nature journals by 346,354 unique authors were analyzed. The acquired data set consisted of papers that were published between January 1, 2019, and August 2, 2020. The proportion of female first authors publishing in the biomedical field during the pandemic dropped by 9.1%, on average, across disciplines (expected arithmetic mean *y_est_*=0.39; observed arithmetic mean *y*=0.35; standard error of the estimate, *S_est_*=0.007; standard error of the observation, *σ_x_*=0.004). The impact was particularly pronounced for papers related to COVID-19 research, where the proportion of female scientists in the first author position dropped by 28% (*y_est_*=0.39; *y*=0.28; *S_est_*=0.007; *σ_x_*=0.007). When looking at the last authors, the proportion of women dropped by 7.9%, on average (*y_est_*=0.25; *y*=0.23; *S_est_*=0.005; *σ_x_*=0.003), while the proportion of women writing about COVID-19 as the last author decreased by 18.8% (*y_est_*=0.25; *y*=0.21; *S_est_*=0.005; *σ_x_*=0.007). Further, by geocoding authors’ affiliations, we showed that the gender disparities became even more apparent when disaggregated by country, up to 35% in some cases.

**Conclusions:**

Our findings document a decrease in the number of publications by female authors in the biomedical field during the global pandemic. This effect was particularly pronounced for papers related to COVID-19, indicating that women are producing fewer publications related to COVID-19 research. This sudden increase in the gender gap was persistent across the 10 countries with the highest number of researchers. These results should be used to inform the scientific community of this worrying trend in COVID-19 research and the disproportionate effect that the pandemic has had on female academics.

## Introduction

As of the date of this writing, the COVID-19 pandemic has claimed hundreds of thousands of lives worldwide and disrupted almost all aspects of human society. The socioeconomic impacts of the pandemic are yet to be assessed and the impending economic crisis and recession are becoming evident [1-3]. During recessions, men are more likely to lose their jobs, as men work in industries that are heavily affected by the slowdown in economic activity, such as manufacturing and construction. Compared to previous economic crises, the current crisis has disproportionately affected female workers [4-11]. One of the reasons for such disparity is women’s overrepresentation in occupations in industries that are most affected by the closures and movement restrictions imposed by public health policies, such as restaurants and hospitality. Another large part of the gender disparity is related to the unequal division of labor in the household, as women are traditionally expected to continue to devote more time to childcare and domestic chores than their partners [4]. In the case of dual-earner, heterosexual married couples with children, the partners have unequally adjusted their work time during the pandemic. Mothers with young children have reduced their work hours 4 to 5 times more than fathers, which contributes to the increased gender gap in earnings [5]. Working mothers affected by the unequal distribution of working hours and the additional burden of domestic chores have reported lower work productivity and job satisfaction than men [6].

Stay-at-home orders, lockdowns, and school closures have affected scientists as well, especially those caring for children or other family members [12,13]. Female scientists reported that their ability to devote time to their research has been substantially affected, and the impact is most pronounced for female scientists with young dependents [14]. The sudden shift in daily activities makes it hard to balance between increasing professional requirements and childcare.

As a result, the research productivity of female scientists appears to have decreased [15-18]. Early evidence suggests that the proportion of publications with female authors is lower during the pandemic with the evident gendered authorship disparities in journal submissions [19,20]. Reports from journal editors in the fields of international studies, political science, economics, medicine, and philosophy indicate that the proportion of submissions authored by women has dropped in most cases [21]. Even though female academics are still submitting manuscripts for publication during the crisis, they are submitting less of their own work than men [22].

A similar effect has been observed with publications on preprint servers. The proportion of female authors publishing on the most popular economics preprint servers is lower than expected [23,24], with only 14.6% of female authors; comparably, they usually make up about 20% of the authors in these databases. Similarly, women publish less in other disciplines, such as physics, earth science, and sociology [25]. In regard to medical and related sciences, on top of the exacerbated gender disparity in publishing during the pandemic, the proportion of female scientists publishing research specifically about COVID-19 is much lower than expected, by almost 23% [25-27].

Motivated by ongoing research efforts, we expand on the previous research by analyzing a large bibliographic data set in the biomedical field; we also employ different modeling techniques that can further improve our understanding of this phenomenon. The aim of this study is to quantify how the COVID-19 crisis exacerbates the gender gap in scientific publishing in the biomedical field.

## Methods

### Data

Bibliometric data on published papers were collected from three separate sources:

1. The bioRxiv repository contains 51,171 papers and 225,110 authors; Rxivist is the application programming interface (API) provider for bioRxiv publications [28].
2. The medRxiv repository contains 8845 papers and 52,364 authors; data are scraped directly from medrxiv.org.
3. The Springer-Nature repository contains 19,525 papers and 91,257 authors; data from 62 journals are collected using the Springer-Nature OpenAccess API. Springer-Nature data include high-impact journals, such as Nature Genetics, Nature Medicine, and Nature Immunology, as well as multiple BMC journals, such as BMC Bioinformatics and BMC Genomics.

We included the data from all journals in the biomedical field for which Springer-Nature provides data. A complete list of journals used in the analysis is available in Table S1 of Multimedia Appendix 1. All the papers from the data set were published between January 1, 2019, and August 2, 2020. The earliest publication on medRxiv is from June 25, 2019.

For each source, we collected the relevant metadata. For each paper in bioRxiv and medRxiv, we kept the *date* of publishing. For Springer-Nature journals, we kept the date of manuscript submission, which is the most comparable date to publication dates in bioRxiv and medRxiv. We additionally stored the *title* and the *abstract* of each paper as well as the scientific discipline of each paper. There were a total of 112 scientific disciplines represented in the data, and each paper belonged to a single discipline. The complete list of all disciplines is provided in Table S2 in Multimedia Appendix 1. For each author, we preserved the *name*, *affiliation*, and the *authorship order*. We removed the papers with group authors, such as scientific consortiums and projects (~0.1% of all papers), as they do not represent individuals. We used socioeconomic data on countries, including their respective gross domestic product (GDP) per capita provided by Our World in Data [29].

### Identifying Authors’ Genders

To infer each author's gender from their name, we used a state-of-the-art tool, namely the genderize.io API [30]. Given an input name, the model returns a gender and a confidence score between 0.5 and 1. The uncertainty is greater for Asian names, which often are not gender specific [31]. We filtered out all authors for which the confidence scores were lower than 0.8. Overall, 19% of names yielded a score below this threshold, with Chinese and Korean names topping the ranking at 54% and 41%, respectively.

In our data set, we identified the most likely gender of 466,836 authors in total. Out of these, the gender of 348,506 (74.7%) unique authors (214,095 male [61.4%] and 134,411 female [38.6%]) could be inferred with high accuracy, with confidence scores from genderize.io higher than 0.8.

### Identifying Authors’ Countries

To identify each author’s country in the bioRxiv and medRxiv data sets, we first located a toponym in each author's affiliation and assigned to it the most likely country code. If there was no toponym, we queried the Global Research Identifier Database, found the institution with the most similar name, and assigned the institution’s country to that author. Additionally, we manually checked the location of the most common affiliation names from the data set that covered most of the authors. The countries of approximately 80% of all authors were determined using this method. The countries of the authors in the Springer-Nature data set were already provided by the API.

### Identifying COVID-19 Papers

The papers that dealt specifically with COVID-19 and similar topics were identified by the set of keywords that appeared in their titles or abstracts.

### Calculating the Differences Between the Expected and Observed Proportions

To measure the discrepancy between the expected and observed proportions of female authors, we first established baselines, which were the expected proportions of female researchers that appeared as authors of publications. The expected proportions were calculated using the ordinary least squares (OLS) model and historical data from January 2019 to March 2020 (see the Model section). We then calculated the true observed proportions of female authors who published during the COVID-19 pandemic in 2020 and compared it to the expected baselines. The error for the predicted value was the mean standard error of the prediction. The error of the observed value was calculated as the standard error of the mean: *SE* = *σ/√n*. The percentage change is calculated as *diff* = (*f^exp^ – f^obs^*)*/f^exp^*, where *f^exp^* is the expected proportion and *f^obs^* is the observed proportion. The errors for the percentage change were calculated as the total sum of the errors of predicted and observed values.

### Model

**Figure 1:**
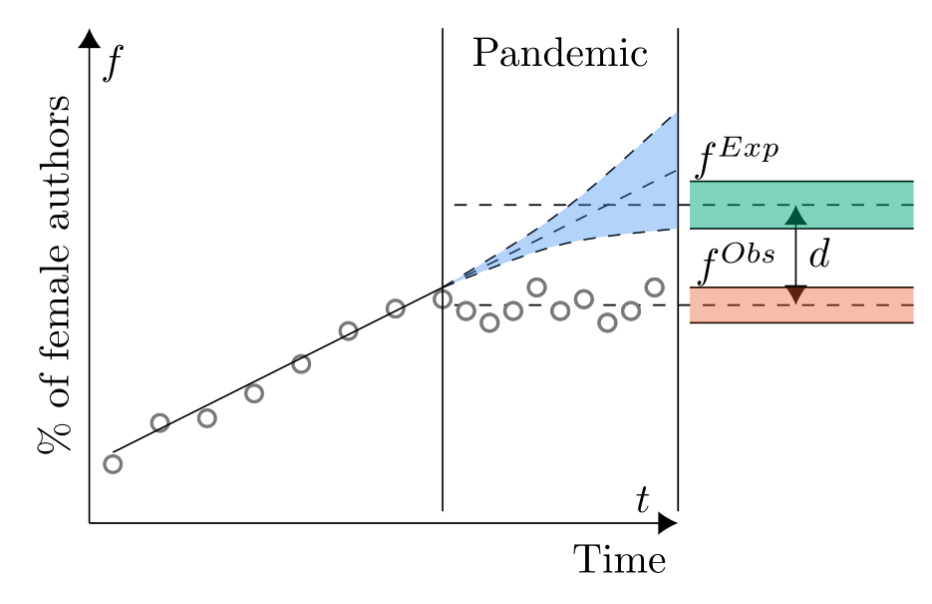
Statistical model. Schematic illustration of the ordinary least squares model used to calculate the expected numbers and proportions. *d*: difference; *f^exp^*: expected proportion; *f^obs^*: observed proportion.

Using historical data from before March 15, 2020, we calculated the proportion of female authors who published each week. We fit an OLS regression model, *f* = *βt* + *c*, where *f* is the proportion of female authors, which serves as a response variable; *t* is the predictor variable—time of publication/submission (to the nearest week); and *β* and *c* are the slope and the intercept, respectively. We fit the separate models depending on the level of disaggregation (country, publisher, etc). The model is illustrated in Figure 1. From the model, we derived the expected fraction, *f^exp^* = *∑f/n*, which is the mean fraction of all predicted values for the observed period and *f^obs^* = *∑f_true_/n*. To estimate the expected number of papers and authors, we used a similar approach, where the response variables were the numbers of papers and authors rather than the proportion of female authors. We used the *statsmodels* [32] package in Python 3.6 (Python Software Foundation) for this purpose.

The OLS model tends to weight all data points equally, regardless of the number of samples. To guarantee the validity of the statistical analysis, we established the conditions under which the data points would be evaluated. The number of data points used to fit the OLS model before March 2020 and the number of data points after March 2020 were at least 10 each. This way, we limited the impact of small-sample observations that could skew the estimate.

We additionally evaluated the model by applying the generalized linear model with binomial errors and a logit link function, as the OLS model could overestimate the proportions in binary variables. Both models performed similarly, and the OLS model did not provide any out-of-norm estimates. For the sake of better interpretability and consistency with modeling the nominal number of authors and papers, we decided to use the OLS model.

To better capture the productivity of the population, we counted each publication from each author separately, effectively modeling the proportion of papers authored by the population of female authors. Considering that multiple authorships in the observed period were relatively rare (<5% of all first authors and <10% of all last authors had >1 paper), we considered each authorship independently.

### Regression Discontinuity Design

To estimate the potential causal effects of the pandemic on the proportion of female researchers, we devised a typical nonparametric regression discontinuity design (RDD) with a local linear regression in time, with the following general form:

*Y* = *α* + *τD* + *β_1_*(*X* – *c*) + *β_2_D*(*X* – *c*) + *ε*

where *c* is the treatment cutoff and *D* is a binary variable equal to 1 if *X* ≥ *c*. In our case, we assumed that the date *c* of a policy change was mid-March 2020. For all dates *t* > *c*, the unit was treated, and for all dates *t* < *c*, the unit was not. This regression discontinuity setup used time-series data and, in this case, weekly observations, both globally and on a country level. By comparing observations lying closely on either side of the temporal threshold, we estimated the average treatment effect. We made sure to focus on observations not too far in time from the threshold, avoiding potential bias from unobservable confounders [33].

The falsification, or placebo, tests were performed by using fake cutoffs before and after mid-March 2020 and comparing the treatment effect. We identified the optimal cutoff point *c*_0_ as the point in time when the treatment effect was the most prominent, *c*_0_ = *c*|max (|*τ*|). The RDD was implemented using the *rdd* package in Python [34].

### Data Availability and Reproducibility

The data and source code for reproducing the results are available at GitHub [35].

## Results

### The Gender Gap in Research During the COVID-19 Pandemic

Overall, during the pandemic, scientists posted papers on preprint servers at an increasing rate. On average, we observed 31.2% more papers than expected and a 41.6% increase in the number of authors (39.2% increase for females and 42.9% increase for males). Despite the absolute increase in the numbers of papers and authors across publishers (see Figure S1 and Tables S3-S5 in Multimedia Appendix 1), the proportion of female authors was lower than expected.

In biology, medicine, and related disciplines, the most active contributors are usually listed first. The author listed last is the most senior author, typically the head of the lab. To address the high variability of the number of authors on the publications (*μ*=7.4, *σ*=9.2), we analyzed the proportion of women, separately, who appeared as the first author, the last author, an author regardless of authorship order, and the solo author. Additionally, we performed a separate analysis on the papers with topics that were directly related to COVID-19 (see Table 1).

**Table 1:** The expected and observed proportions of female authors disaggregated by the order of authorship and the topic.

Author order and paper topic		Expected proportion		Observed proportion		Drop, %
		*y_est_^a^*	*S_est_^b^*	*y^c^*	*σ_x_^d^*	
**First**						
	All	0.389	0.007	0.353	0.004	9.142
	COVID-19	0.389	0.007	0.280	0.007	28.031
	Non-COVID-19	0.389	0.007	0.380	0.004	2.372
**Last**						
	All	0.257	0.005	0.236	0.003	7.961
	COVID-19	0.257	0.005	0.209	0.007	18.812
	Non-COVID-19	0.257	0.005	0.246	0.003	4.416
**Any**						
	All	0.354	0.003	0.348	0.002	1.578
	COVID-19	0.354	0.003	0.341	0.009	3.530
	Non-COVID-19	0.354	0.003	0.351	0.002	0.934
**Solo**						
	All	0.210	0.030	0.137	0.008	34.586
	COVID-19	0.210	0.030	0.137	0.023	34.514
	Non-COVID-19	0.210	0.030	0.168	0.013	19.802

^a^*y_est_*: the arithmetic mean of the estimate.

^b^*S_est_*: the mean standard error of the estimate.

^c^*y*: the arithmetic mean of the observation.

^d^*σ_x_*: the standard error of the mean of the observation.

**Figure 2:**
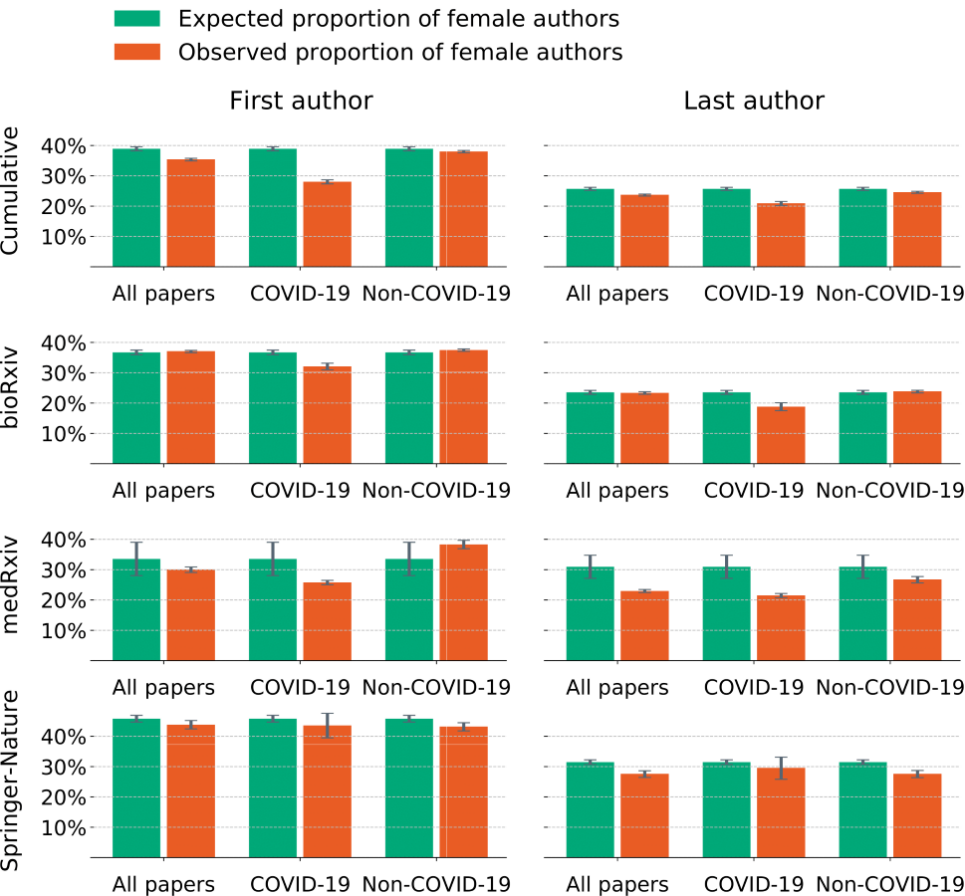
Comparison of the expected and observed proportions of female authors that published during the COVID-19 pandemic. Green bars represent the expected proportion of female authors, estimated by the ordinary least squares model from the historical data from 2019. Orange bars represent the observed proportion of female authors that published during the COVID-19 pandemic. The standard errors of the aggregate analyses are represented as the vertical lines on top of the bars. The papers are divided by topic into three groups: (1) all papers from the data set, (2) papers that deal directly with COVID-19 and related topics, and (3) papers that are not about COVID-19 or related topics. The first row shows the results from all publishers combined. The following rows represent the results for each publisher separately.

The aggregate results suggest that the proportion of female authors publishing on all topics as the first author decreased by 9.1% (expected arithmetic mean *y_est_*=0.38; observed arithmetic mean *y*=0.35; standard error of the estimate, *S_est_*=0.007; standard error of the observation, *σ_x_*=0.004). The percentage drop became unusually prominent when we analyzed the papers about COVID-19. The proportion of female scientists who wrote on COVID-19-related topics as the first author was lower by 28% (*y_est_*=0.38; *y*=0.27; *S_est_*=0.007; *σ_x_*=0.007). When considering the last authors, the proportion of women writing about COVID-19 decreased by 18.8% (*y_est_*=0.25; *y*=0.2; *S_est_*=0.005; *σ_x_*=0.007). However, when we focused only on papers that did not deal with COVID-19, we saw a smaller change both for the first author (2.3%) and the last author (4.4%). The proportion of women publishing papers on topics other than COVID-19 on medRxiv increased by 14%, on average. The results are shown in Table 1 and are illustrated in Figure 2. The expected proportions are plotted as the green bars, and the true proportions are plotted in orange. The standard errors were relatively small (see Methods section for details about the error calculation).

Additionally, we focused our analysis on the papers with a single author and discovered an even greater disparity. We observed 34.5% (*y_est_*=0.21; *y*=0.13; *S_est_*=0.03; *σ_x_*=0.008) fewer female solo authors during the pandemic who published on all topics across the platforms (see Figure S2 in Multimedia Appendix). A similar disparity appeared in case of the solo authors publishing papers about COVID-19. Note that only 3.2% (2551/79,528) of all papers were authored by a single author, hence, the relatively large standard errors of both the estimate and the observation, especially for papers published on medRxiv. The effect still exists, although much less prominently, when we observed all authors regardless of the order of authorship. More detailed information is provided in Table S3 in Multimedia Appendix 1.

The results suggest that the aggregate gender disparity in academia during the pandemic was due to the increased publication rate of papers about COVID-19 authored by men. To further explore this possibility, we tracked the individual publication records and calculated the probability that the author would publish work about COVID-19. Around 3.7% of men who had publication records in our data set would publish at least one paper about COVID-19, compared to ~2.2% of women. Men who already had a publication before the pandemic were 37% more likely to publish a paper about COVID-19. This suggest that women are getting excluded from critical research about COVID-19.

When disaggregated by publisher, the relative drop in the proportion of female first authors for COVID-19-related research was 12.6%, 23.2%, and 2.1% for bioRxiv, medRxiv, and Springer-Nature journals, respectively (see Figure 2). A similar disparity was observed for last authors, with a relative drop of 20.1%, 30.8%, and 23.6%, and for authors regardless of the authorship order, with a relative drop of 2.2%, 10.7%, and 16.1% for bioRxiv, medRxiv, and Springer-Nature journals, respectively. In the case of solo papers, the average drop across the platforms was 34.5%. The proportion of females publishing on topics other than COVID-19 remained within the standard error of the estimate, without strong evidence of decrease. Note the large standard errors in the estimated proportion of women publishing COVID-19-related papers in Springer-Nature journals due to the lack of data. Only published papers have metadata available through the Springer-Nature API, and many papers submitted during the pandemic that will ultimately be published have not yet been accepted and published (see Methods section).

Additionally, we checked whether there was a significant change in the proportion of women authors that occurred in mid-March 2020. To test the hypothesis, we performed an RDD analysis in time (see Methods section). We estimated a vertical discontinuity of the proportion of women over time by the coefficient *τ* at the cutoff point *c*_0_ = March 15, 2020. For all the papers, regardless of the topic, we obtained *τ*=–0.008 with *P*=.03. However, when considering only the papers about COVID-19, the discontinuity became clearer with *τ*=–0.049 and *P*<.001. To assert the robustness of our model, we performed a placebo test (see Methods section) to confirm that the discontinuity was likely aligned with the start of the pandemic, and that it happened at or around March 2020 and not during any time in 2019. When RDD analysis was performed at the country level, we confirmed that, for most countries, the cutoff threshold fell between mid-March and mid-April 2020 (see Table S6 in Multimedia Appendix 1). The RDD analysis suggests that there was a drop in the proportion of female authors at the beginning of April 2020 that was more significant than any other fluctuation that occurred in 2019 or after April 2020.

Further, we checked whether we could confidently use the proportion of women who published before the pandemic as the reference to estimate the proportion of women who published papers specifically about COVID-19. A hypothesis is that before the pandemic, women were less likely to be represented in the scientific disciplines that would produce COVID-19 research. To check this hypothesis, we first performed a chi-square test on the distribution of disciplines involved in COVID-19 research. We discovered that some disciplines, such as infectious diseases, epidemiology, public health, and global health, were overrepresented (*P*<.001). Then, we tested whether the proportions of women in COVID-19 disciplines were significantly different from non-COVID-19 disciplines. By performing the Kolmogorov-Smirnov test, we compared the distributions of the proportion of women across two groups of disciplines and we obtained *P*=.84. We conclude that the two groups were sampled from populations with the same distributions, and we can be confident that we can use the data on the proportion of women from before the pandemic to model the proportion of women that publish about COVID-19.

### Some Trends During the Pandemic

To assess the temporal trends during the pandemic, we built the linear model *f(t)* = *α* + *βt* + *ε*, where *f*(*t*) is the proportion of female scientists, and *t* is the time in weeks after mid-March 2020. The regression coefficient *β* is used to quantify the trend. We did not identify a significant change in the proportion of female first authors (see Table 2). However, we observed a small but significant increase in the proportion of female scientists appearing as the last author (*β*=.001, *P*=.002) and as an author regardless of authorship order (*β*=.001, *P*<.001). An even stronger positive trend was observed for the COVID-19-related research for the last author (*β*=.003, *P*<.001) and for an author regardless of authorship order (*β*=.005, *P*<.001) (see Table S7 in Multimedia Appendix 1).

**Table 2:** Parameters of the linear model of the proportion of female authors over time during the pandemic.

Paper topic	First author		Last author		All authors		Solo author	
	*β^a^*	*P* value	*β*	*P* value	*β*	*P* value	*β*	*P* value
All	–.002	.08	.002	.002	.001	<.001	.000	>.99
COVID-19	.001	.28	.003	<.001	.005	<.001	–.005	.12
Non-COVID-19	–.002	.02	.001	.03	.000	.17	.004	.13

^a^*β* is a regression coefficient.

### Country-Level Analysis

**Figure 3:**
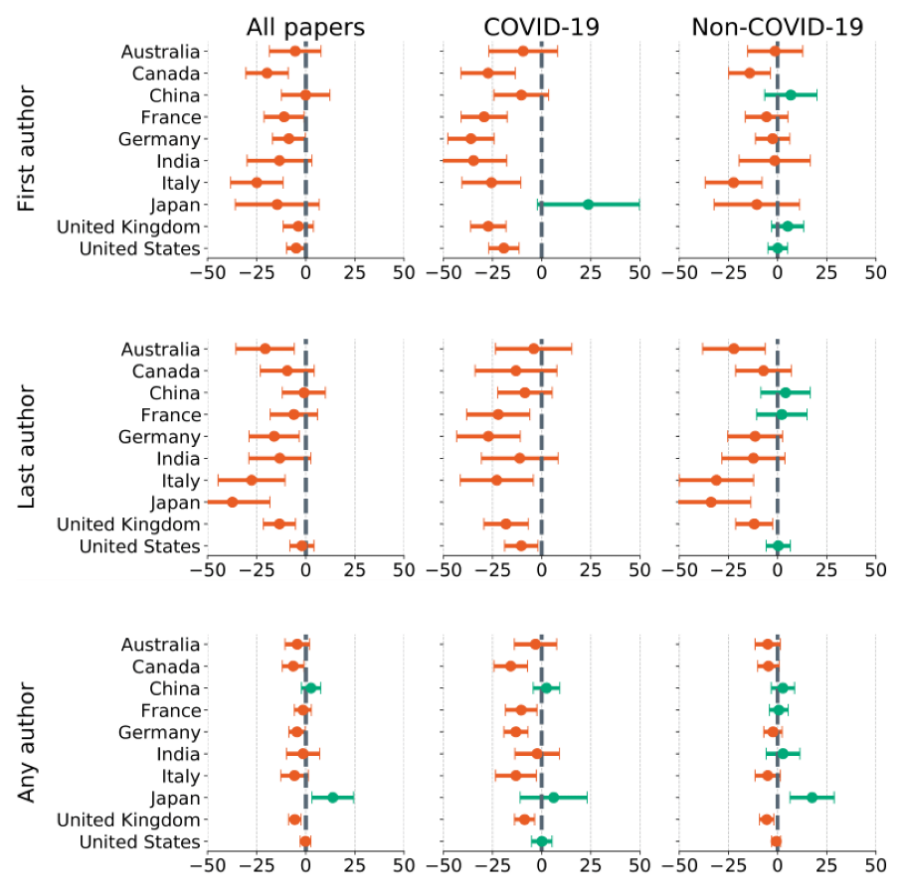
Percentage drop in proportion of female authors during the pandemic across countries. Orange points mark the percentage decrease in proportion of female authors; green points mark the increase. Horizontal lines represent standard errors. The analysis is divided by topic into three groups: (1) all papers from the data set, (2) papers that deal directly with COVID-19 and related topics, and (3) papers that are not about COVID-19 or related topics. Missing points indicate insufficient sample size.

We identified the most likely country of the authors based on their affiliations (see Methods section) and measured the difference between the expected and observed proportions of female authors during the pandemic. Figure 3 shows the pandemic-related gender gap across the countries with the largest share of authors. The values represent percentage differences between the expected and observed fractions of female authors publishing in bioRxiv, medRxiv, and selected Springer-Nature journals between March and August 2020. Points to the left of the midline (orange) represent countries with less than expected fractions of female authors, and points to the right of the midline (in green) represent countries an increase in the fractions of female authors. The left-hand plots are for all papers regardless of topic, the middle plots are only for COVID-19-related papers, and the right-hand plots are only for papers that are not related to COVID-19. More detailed information is provided in Tables S8-S10 in Multimedia Appendix 1.

A significant drop in the proportion of female first authors was consistent across the countries. Regardless of the topic, we observed a 24.9% drop in Italy (*y_est_*=0.526; *y*=0.395; *S_est_*=0.046; *σ_x_*=0.024), followed by Canada (19.7%), Sweden (15.9%), Japan (14.5%), India (13.4%), and France (11.1%). For research dealing explicitly with the topic of COVID-19 (see Figure 3, middle panels), we observed a greater gender gap than with papers on other research topics (see Figure 3, right-hand panels). In Germany, for example, the relative drop in the proportion of female first authors was 36% (*y_est_*=0.39; *y*=0.25; *S_est_*=0.02; *σ_x_*=0.027), indicating that male scientists affiliated with German institutions are publishing disproportionately more than their female colleagues about COVID-19. Similar considerations applied to India, France, Italy, Great Britain, Canada, and the United States. The opposite was true for Japan, where the proportion of women publishing about COVID-19 as the first author increased by 23.7%. A similar disparity applied to the last authors (see Figure 3, second row). Missing points indicate that there were not enough data from the pandemic period to calculate the observed mean.

When we observed the proportion of female authors regardless of the authorship order, the drop became less prominent but still consistent across the countries. For example, in Canada, a drop in the proportion of female authors for COVID-19-related papers was 15.7% (*y_est_*=0.387; *y*=0.318; *S_est_*=0.014; *σ_x_*=0.018), with similar-sized drops for Germany, Italy, Great Britain, and France (see Figure 3, third row). On the other hand, there was an increase of 6% in the proportion of female authors writing about COVID-19 in Japan (*y_est_*=0.155; *y*=0.164; *S_est_*=0.010; *σ_x_*=0.017). The increase in the proportion of female authors in China (2.4%) was within the margin of error.

The gender gap for non-COVID-19-related research (see Figure 3, right-hand panels) was found to exist during the pandemic, but it is smaller than for COVID-19 research. Again, we observed stark differences between countries, with the proportion of female first authors publishing during the pandemic significantly decreasing in Italy, Canada, Japan, and France. Note that the plots for the single-author papers are missing, as the samples became too small when disaggregated by country.

**Figure 4:**
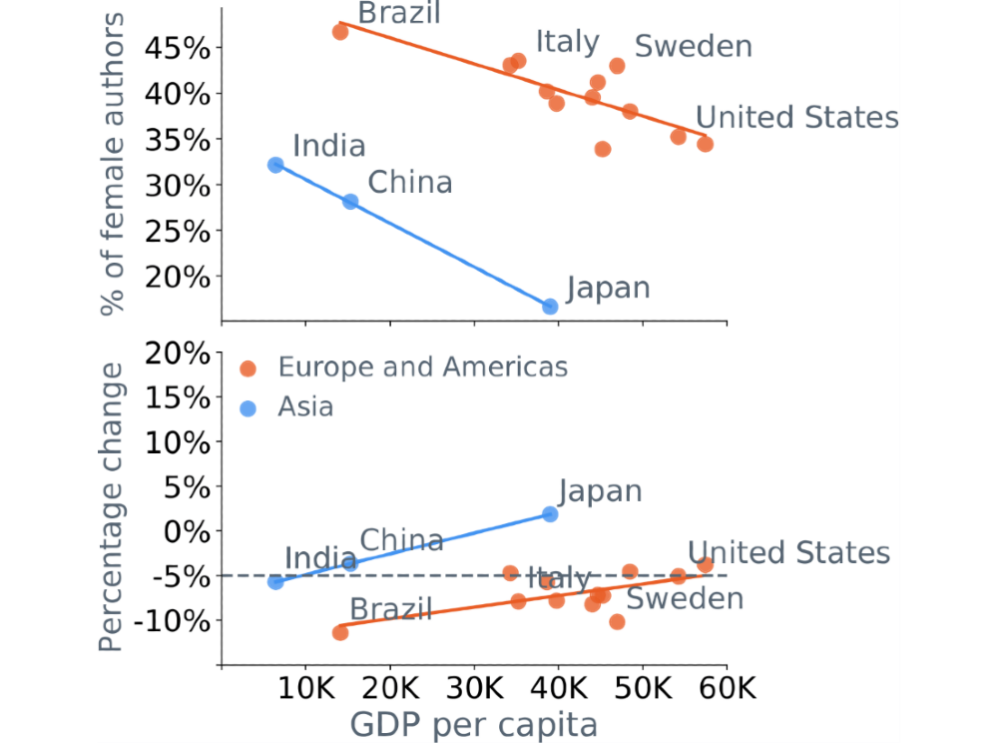
Gender disparity in research and gross domestic product (GDP). The proportion of women active in research is higher in countries with lower per capita GDP (upper). The proportion of female authors of research articles decreased more than expected in countries with lower per capita GDP (lower).

Further, we explored whether there were any commonalities among the countries with respect to the participation of women in research. Figure 4 illustrates the proportion of female authors (upper panel) and the percentage change in the proportion of female authors (lower panel) as a function of GDP. When disaggregated by region, we observed that the wealthier countries—those with higher per capita GDP—had proportionally fewer female researchers, with Asian countries exhibiting the most pronounced gender disparity. However, the countries with a higher GDP per capita demonstrated a smaller drop in the proportion of women publishing during the pandemic.

## Discussion

### Principal Findings

We analyzed bibliographical data from biomedical preprint servers and Springer-Nature journals and showed that the fraction of women publishing during the COVID-19 pandemic dropped significantly across disciplines and research topics. Since the announcement of the global pandemic and the start of lockdowns, we observed a drop of 9.1% in the number of women publishing biomedical scientific papers as the first author. Women were significantly excluded from COVID-19-related research, as we measured a 28% drop in female first authors in that area of research. This confirms some earlier suggestions that female first authors contributed less to COVID-19 studies than to research in other areas [25]. Women remain underrepresented, even though we observed an increased publishing rate for both genders during the pandemic. A similar disparity can be observed for last authors as well as solo-authored papers. The increased gender gap in publishing is persistent across the 10 countries with the highest number of researchers.

For papers on topics other than COVID-19, we did not observe this high discrepancy, and, in the case of medRxiv, we observed more women than projected by the model. The overall gender disparity in research during the pandemic was mostly driven by the higher publication rate of papers on COVID-19 and related topics. It seems that such research is conducted disproportionately by men, as male authors are more likely to appear in first author positions on papers posted on preprint servers and published in peer-reviewed journals.

It appears that the most significant drop in proportion of female authors happened early in the pandemic. The proportion of women has been increasing gradually for some authorship categories. Note that the observed gradual increase is statistically significant but is very slow. One can think that a possible explanation for such a sudden drop and a subsequent gradual increase is that most of the COVID-19 papers published early during the pandemic were various epidemic models focusing on cases and death counts. Many of the authors’ affiliations were departments of engineering, mathematics, and physics, which might have a different proportion of women than the population of scientists in biology and medicine. Since research in the biomedical field usually takes longer to conduct and publish, it could lead to a shift in the gender distribution later. However, this argument does not explain the phenomenon entirely, as the base gender gap in science, technology, engineering, and mathematics fields is not higher than in biology [31]; therefore, future publications from biologists are not expected to narrow the gender gap. On the contrary, they might even increase it.

Another likely explanation of a sudden drop in the proportion of female authors is that caregiving demands have exploded during the pandemic, and these have mostly fallen on women [12,36,37]. These include childcare demands [38], elderly care, and other kinds of domestic and emotional labor. Sudden lockdowns and other preventive measures unevenly increased the burden on certain populations, causing the productivity of female scientists to decrease. As the world started fighting off the pandemic, people got used to the “new normal” and scientists started returning to their routines. That can partially explain the gradual increase in the proportion of female authors. Nevertheless, further research and more time is needed to investigate the reasons for such a sudden drop and gradual revival of the proportion of papers published by female scientists during the pandemic.

The global pandemic has touched almost every nation on the planet. Countries, however, responded differently in containing the spread of the disease. The variability of the measures and their timing, combined with differences in cultural norms and outbreak severity, have had a variable impact on researchers across the world. Country-level analysis better reveals global trends, as the aggregate data can be skewed by countries with a disproportionately large number of publications, such as the United States, which represents almost 29% of all authors in the data set (see Table S11 in Multimedia Appendix 1). Additionally, our analysis can reveal regional, political, and cultural differences between the nations. It is known that gender disparities in research are strongly associated with a country’s wealth [39]. The wealthier countries—those with higher per capita GDP—have proportionally fewer female researchers, with Asian countries exhibiting the most pronounced gender disparity. However, the countries with higher GDP per capita were more resilient to the effects of COVID-19 on gender imbalance. In addition, wealthier countries showed a smaller pandemic-related drop in women’s participation in research than poorer countries, with wealthier Asian countries experiencing an increase in the proportion of active female researchers. This suggests that women experience bigger life disruptions in poorer countries, which affects their productivity. Additionally, women are more excluded from COVID-19 research in poorer nations. This certainly should not imply any purpose or deliberate action, but rather the disproportionate variations in the social environments across nations, caused by the various expectations for the female members of households.

### Implications

Gender imbalances in academia have been evident historically and still persist today. Various measures of research output, including the proportion of authors, fractionalized authorships [40], tenure decisions, and number of research grants [41], indicate the significant gender gap that is observed worldwide. For the past 60 years, we have witnessed an increase in participation by women in science across scientific disciplines [31] and lower levels of discrimination [42], demonstrating that the gender gap is shrinking over time [43]. Thus, a sudden drop in women’s research output in biomedical research about COVID-19 appears as a surprising reverse trend.

The factors that led to such extreme and consistent differences in the proportion of female scientists can be numerous. The already existing barriers for female participation in science vary across countries. In some nations, men are more favorably placed than women [44-47] and can be more likely to receive quick funding for COVID-19-related research. Additionally, traditional gender norms differ and can affect the genders differently. Caregiving demands have mostly fallen on women. At the same time, new challenges bring new opportunities, and men who are likely to engage in more aggressive self-promotion [48,49] and pursue careers more forcefully [43] can be motivated to push for faster publication. Identifying the exact reasons for an increased gender gap can be an important topic for future studies.

The global pandemic caused this unforeseen crisis that will most certainly affect academia. All the difficulties female scientists faced previously may possibly be exacerbated by the extended lockdowns and sudden shift in work-life dynamics. It is important to understand the impact of such an extraordinary circumstance on the scientific community that will disproportionately affect research outputs as well as prospects for tenure and promotions [21]. Future research evaluation practices should be informed by our findings to account for and mitigate the penalizing effects that COVID-19 is having on female researchers.

### Strengths and Limitations of the Study

The strengths of our study include the use of a relatively large and diverse data set from three different publishing platforms. The focus on preprint papers allows for the assessment of the observed effects in a timely manner. We focused on a structured and rigorous statistical analysis, making sure that the results are significant. The data and the code to reproduce the results are available.

Potential limitations warrant consideration. First, the gender of a publication’s author can be wrongly identified. Even though we excluded the results that had a low confidence, a small fraction of the authors could have been misgendered. Additionally, we acknowledge that automated gender classifiers do not recognize the various nonbinary gender identities [50], and we assigned the gender label based on the historical distribution of typical male and female names. As awareness of the nature of gender and identity shift, so may the number of researchers who do not identify within the binary categories of male and female. Such researchers face additional layers of discrimination that our study does not consider. While we understand that binary gender can be an oversimplification that can introduce some amount of bias and inaccuracy, the problem that we highlight hopefully can bring some attention to the multifaceted issue of gender, identity, and discrimination. Second, the algorithm that identified the authors’ countries relies on recognizing the names of the toponyms in the names of the authors’ affiliated institutions. Even though we made sure that the most popular institutions were properly localized and we optimized the localization resolution, some errors are possible. Third, throughout the paper, the word “productivity” was used to refer to the rate of publication output of scientists in terms of publications per week and it did not capture changes to scientists’ other inputs. For example, female scientists appearing less productive in terms of publications per week may simply reflect that they were not able to spend as much time on their research (ie, hours worked were not captured). We are aware that there are other preprint servers and journals that publish papers in the biomedical field. By analyzing the data from the two largest preprint servers and the largest publisher of peer-reviewed papers, we aimed to cover a representative sample of papers and authors from the field. Finally, our analysis was focused on the first 6 months of the pandemic and might not accurately evaluate the effects that can be observed later in the pandemic.

### Conclusions

Our findings documented a decrease in the proportion of female authors in the biomedical field who published research papers during the global pandemic. This effect was particularly pronounced for papers related to COVID-19, indicating that women are producing fewer publications related to COVID-19 research. A sudden increase in this gender gap was persistent across the 10 countries with the highest number of researchers. The results should be used to inform the scientific community of this worrying trend in COVID-19 research and the disproportionate effect the pandemic has had on female academics’ research outputs.

## Appendix

Multimedia Appendix 1 Supplementary materials.

## Abbreviations

API: application programming interface
DARPA: Defense Advanced Research Projects Agency
GDP: gross domestic product
OLS: ordinary least squares
RDD: regression discontinuity design
