# Supplementary material for: Gender Disparity in the Authorship of Biomedical Research Publications During the COVID-19 Pandemic: Retrospective Observational Study
Source: J Med Internet Res. 2021 Apr 12;23(4):e25379. doi: 10.2196/25379 (PMC8043146; doi:10.2196/25379)
Supplement: Multimedia Appendix 1 [file jmir_v23i4e25379_app1.pdf]

Multimedia Appendix for

Gender disparity in the authorship of biomedical research publications during the COVID-19 pandemic

Goran Murić, Kristina Lerman and Emilio Ferrara

Emilio Ferrara
E-mail: emiliofe@usc.edu

This PDF file includes:

Figs. S1 to S2
Tables S1 to S11

Table S1. The complete list of Springer-Nature Journals used for the analysis

Journals
BMC Anesthesiology
BMC Biochemistry
BMC Bioinformatics
BMC Biology
BMC Biotechnology
BMC Cancer
BMC Cardiovascular Disorders
BMC Complementary and Alternative Medicine
BMC Dermatology
BMC Developmental Biology
BMC Ecology
BMC Emergency Medicine
BMC Endocrine Disorders
BMC Evolutionary Biology
BMC Family Practice
BMC Gastroenterology
BMC Genetics
BMC Genomics
BMC Geriatrics
BMC Health Services Research
BMC Immunology
BMC Infectious Diseases
BMC International Health and Human Rights
BMC Medical Education
BMC Medical Ethics
BMC Medical Genetics
BMC Medical Genomics
BMC Medical Imaging
BMC Medical Informatics and Decision Making
BMC Medical Research Methodology
BMC Medicine
BMC Microbiology
BMC Molecular Biology
BMC Musculoskeletal Disorders
BMC Nephrology
BMC Neurology
BMC Neuroscience
BMC Nursing
BMC Obesity
BMC Ophthalmology
BMC Oral Health
BMC Palliative Care
BMC Pediatrics
BMC Plant Biology
BMC Pregnancy and Childbirth
BMC Psychiatry
BMC Psychology
BMC Public Health
BMC Pulmonary Medicine
BMC Sports Science, Medicine and Rehabilitation
BMC Structural Biology
BMC Surgery
BMC Urology
BMC Veterinary Research
BMC Women's Health
Nature Biomedical Engineering
Nature Biotechnology
Nature Chemistry
Nature Genetics
Nature Immunology
Nature Medicine
Nature Microbiology

Table S2. The list of scientific disciplines within the biomedical fields

	Discipline	Papers	Authors		Discipline	Papers	Authors
1	Neuroscience	9412	39401	57	Evolutionary Biology	184	765
2	Microbiology	5354	28964	58	Palliative Care	171	901
3	Bioinformatics	4629	17754	59	Biology	155	1153
4	Genomics	3695	24051	60	Medical Genomics	143	873
5	Cell-Biology	2896	17044	61	Emergency Medicine	136	672
6	Evolutionary-Biology	2487	9897	62	Complementary And Alternative Medicine	128	515
7	Ecology	2380	10092	63	Medical Imaging	127	539
8	Genetics	2369	17309	64	Psychiatry And Clinical Psychology	123	830
9	Biophysics	2183	8627	65	Psychology	114	526
10	Biochemistry	1995	11158	66	Health Informatics	113	605
11	Molecular-Biology	1992	11921	67	Genetic And Genomic Medicine	111	1219
12	Cancer-Biology	1990	16045	68	Medical Ethics	109	442
13	Public Health	1966	9428	69	Nursing	101	420
14	Immunology	1936	14778	70	Biotechnology	91	481
15	Plant-Biology	1774	8711	71	Cardiovascular Medicine	81	610
16	Developmental-Biology	1524	8266	72	Oncology	79	862
17	Bioengineering	1377	6846	73	Radiology And Imaging	73	518
18	Epidemiology	1348	6864	74	Sports Science, Medicine And Rehabilitation	71	355
19	Cancer	1258	7881	75	Health Policy	69	335
20	Health Services Research	1148	5615	76	Intensive Care And Critical Care Medicine	65	605
21	Systems-Biology	1110	5713	77	Respiratory Medicine	61	615
22	Infectious Diseases (Except Hiv/Aids)	1094	10056	78	Paleontology	61	276
23	Infectious Diseases	1005	5677	79	Occupational And Environmental Health	47	296
24	Physiology	859	5266	80	Health Economics	36	114
25	Musculoskeletal Disorders	811	3543	81	Health Systems And Quality Improvement	35	270
26	Animal-Behavior-And-Cognition	770	2884	82	Clinical-Trials	30	303
27	Pediatrics	646	3253	83	Endocrinology (Including Diabetes Mellitus And...	29	220
28	Plant Biology	642	2621	84	Allergy And Immunology	28	385
29	Pregnancy And Childbirth	620	2877	85	Dermatology	27	152
30	Neurology	578	3544	86	Hiv/Aids	25	190
31	Pharmacology-And-Toxicology	566	3324	87	Developmental Biology	25	103
32	Psychiatry	538	2914	88	Nutrition	23	135
33	Nephrology	530	2890	89	International Health And Human Rights	23	121
34	Veterinary Research	504	2641	90	Pharmacology And Therapeutics	22	158
35	Cardiovascular Disorders	458	2339	91	Obstetrics And Gynecology	20	141
36	Synthetic-Biology	454	2200	92	Rehabilitation Medicine And Physical Therapy	20	129
37	Medical Education	445	2016	93	Primary Care Research	17	141
38	Geriatrics	434	2334	94	Rheumatology	17	120
39	Ophthalmology	430	1446	95	Geriatric Medicine	16	111
40	Pathology	384	2756	96	Hematology	15	174
41	Gastroenterology	368	2093	97	Dentistry And Oral Medicine	14	66
42	Scientific-Communication-And-Education	359	1676	98	Sports Medicine	13	73
43	Public And Global Health	348	2113	99	Addiction Medicine	12	87
44	Oral Health	346	1406	100	Unknown	12	69
45	Anesthesiology	320	1357	101	Sexual And Reproductive Health	10	53
46	Medicine	301	4446	102	Otolaryngology	10	52
47	Pulmonary Medicine	289	1623	103	Pain Medicine	6	34
48	Surgery	285	1403	104	Orthopedics	6	25
49	Zoology	272	1213	105	Toxicology	5	32
50	Medical Genetics	236	1186	106	Palliative Medicine	4	17
51	Medical Research Methodology	230	1143	107	Transplantation	4	14
52	Medical Informatics And Decision Making	225	1110	108	Anesthesia	4	11
53	Women'S Health	223	978	109	Biomedical Engineering	2	42
54	Family Practice	213	1033	110	Chemistry	2	22
55	Endocrine Disorders	210	1068	111	Molecular Biology	2	10
56	Urology	193	1037	112	Obesity	1	7

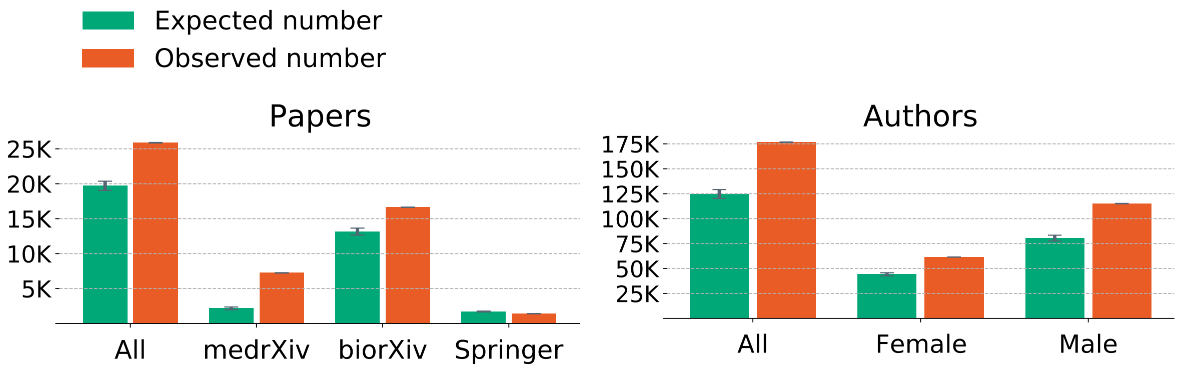

Fig. S1. Number of papers and authors during the COVID-19 pandemic. Green bars are the expected numbers and the orange bars are the actual numbers. We observe high influx of papers on preprint servers and drop of submissions to peer-reviewed journals. The number of authors publishing during the pandemic is higher than expected.

Table S3. The expected and observed proportion of female authors disaggregated by the publisher and the order of authorship

	order	papers	Model $\bar{y}_{est}$	$\bar{S}_{est}$	Observation $\bar{y}$	$\sigma_{\bar{x}}$	% drop
All publishers	First	All	0.389	0.007	0.353	0.004	9.142
		COVID-19	0.389	0.007	0.28	0.007	28.031
		Non-Covid-19	0.389	0.007	0.38	0.004	2.372
	Last	All	0.257	0.005	0.236	0.003	7.961
		COVID-19	0.257	0.005	0.209	0.007	18.812
		Non-Covid-19	0.257	0.005	0.246	0.003	4.416
	Any	All	0.354	0.003	0.348	0.002	1.578
		COVID-19	0.354	0.003	0.341	0.009	3.53
		Non-Covid-19	0.354	0.003	0.351	0.002	0.934
	Solo	All	0.21	0.03	0.137	0.008	34.586
		COVID-19	0.21	0.03	0.137	0.023	34.514
		Non-Covid-19	0.21	0.03	0.168	0.013	19.802
bioRxiv	First	All	0.367	0.008	0.37	0.004	-0.826
		COVID-19	0.367	0.008	0.32	0.011	12.671
		Non-Covid-19	0.367	0.008	0.375	0.004	-2.052
	Last	All	0.235	0.007	0.233	0.004	0.899
		COVID-19	0.235	0.007	0.188	0.013	20.082
		Non-Covid-19	0.235	0.007	0.238	0.004	-1.065
	Any	All	0.342	0.003	0.344	0.002	-0.596
		COVID-19	0.342	0.003	0.334	0.007	2.287
		Non-Covid-19	0.342	0.003	0.345	0.002	-0.82
	Solo	All	0.209	0.035	0.165	0.016	21
		COVID-19	0.209	0.035	–	–	–
		Non-Covid-19	0.209	0.035	0.175	0.018	15.996
medRxiv	First	All	0.335	0.055	0.3	0.009	10.6
		COVID-19	0.335	0.055	0.258	0.007	23.21
		Non-Covid-19	0.335	0.055	0.383	0.014	-14.181
	Last	All	0.309	0.038	0.23	0.005	25.789
		COVID-19	0.309	0.038	0.215	0.007	30.678
		Non-Covid-19	0.309	0.038	0.267	0.011	13.79
	Any	All	0.376	0.016	0.348	0.005	7.519
		COVID-19	0.376	0.016	0.336	0.007	10.794
		Non-Covid-19	0.376	0.016	0.371	0.004	1.385
	Solo	All	–	–	0.145	0.025	–
		COVID-19	–	–	0.114	0.013	–
		Non-Covid-19	–	–	0.269	0.031	–
Springer-Nature	First	All	0.458	0.011	0.438	0.014	4.33
		COVID-19	0.458	0.011	0.435	0.04	4.949
		Non-Covid-19	0.458	0.011	0.431	0.014	5.847
	Last	All	0.315	0.008	0.275	0.011	12.537
		COVID-19	0.315	0.008	0.295	0.036	6.372
		Non-Covid-19	0.315	0.008	0.275	0.012	12.477
	Any	All	0.39	0.006	0.382	0.007	1.997
		COVID-19	0.39	0.006	0.37	0.032	5.022
		Non-Covid-19	0.39	0.006	0.374	0.009	4.131
	Solo	All	0.284	0.04	0.299	0.029	-5.149
		COVID-19	0.284	0.04	–	–	–
		Non-Covid-19	0.284	0.04	0.362	0.04	-27.352

$\bar{y}_{est}$ is the arithmetic mean of the estimate, $\bar{y}$ is the arithmetic mean of the observation
$\bar{S}_{est}$ is the mean standard error of the estimate
$\sigma_{\bar{x}}$ is the standard error of the mean (SEM) of the observation
$p > 0.05$ for all baseline models – no significant temporal trend observed

Table S4. The expected and observed number of authors

	Model		Observation		
	$\sum \bar{y}_{est}$	$\sum \bar{S}_{est}$	$\sum \bar{y}$	$\sum \sigma_{\bar{x}}$	% diff
All	124710.526	176627	4460.152	257.515	41.630
Female	44182.437	61528	1605.054	91.687	39.259
Male	80528.089	115099	2897.689	168.291	42.930

$\bar{y}_{est}$ is the arithmetic mean of the estimate
$\bar{y}$ is the arithmetic mean of the observation
$\bar{S}_{est}$ is the mean standard error of the estimate
$\sigma_{\bar{x}}$ is the standard error of the mean (SEM) of the observation
$p < 0.05$ for all baseline models

Table S5. The expected and observed number of papers

	Model		Observation		
	$\sum \bar{y}_{est}$	$\sum \bar{S}_{est}$	$\sum \bar{y}$	$\sum \sigma_{\bar{x}}$	% diff
All	19706.087	25867	658.846	35.692	31.264
medRxiv	2165.893	7244	198.779	22.482	234.458
bioRxiv	13156.660	16634	495.626	22.691	26.430
Springer-Nature	4770.883	1991	195.950	15.270	-58.268

$\bar{y}_{est}$ is the arithmetic mean of the estimate
$\bar{y}$ is the arithmetic mean of the observation
$\bar{S}_{est}$ is the mean standard error of the estimate
$\sigma_{\bar{x}}$ is the standard error of the mean (SEM) of the observation
$p < 0.05$ for all baseline models

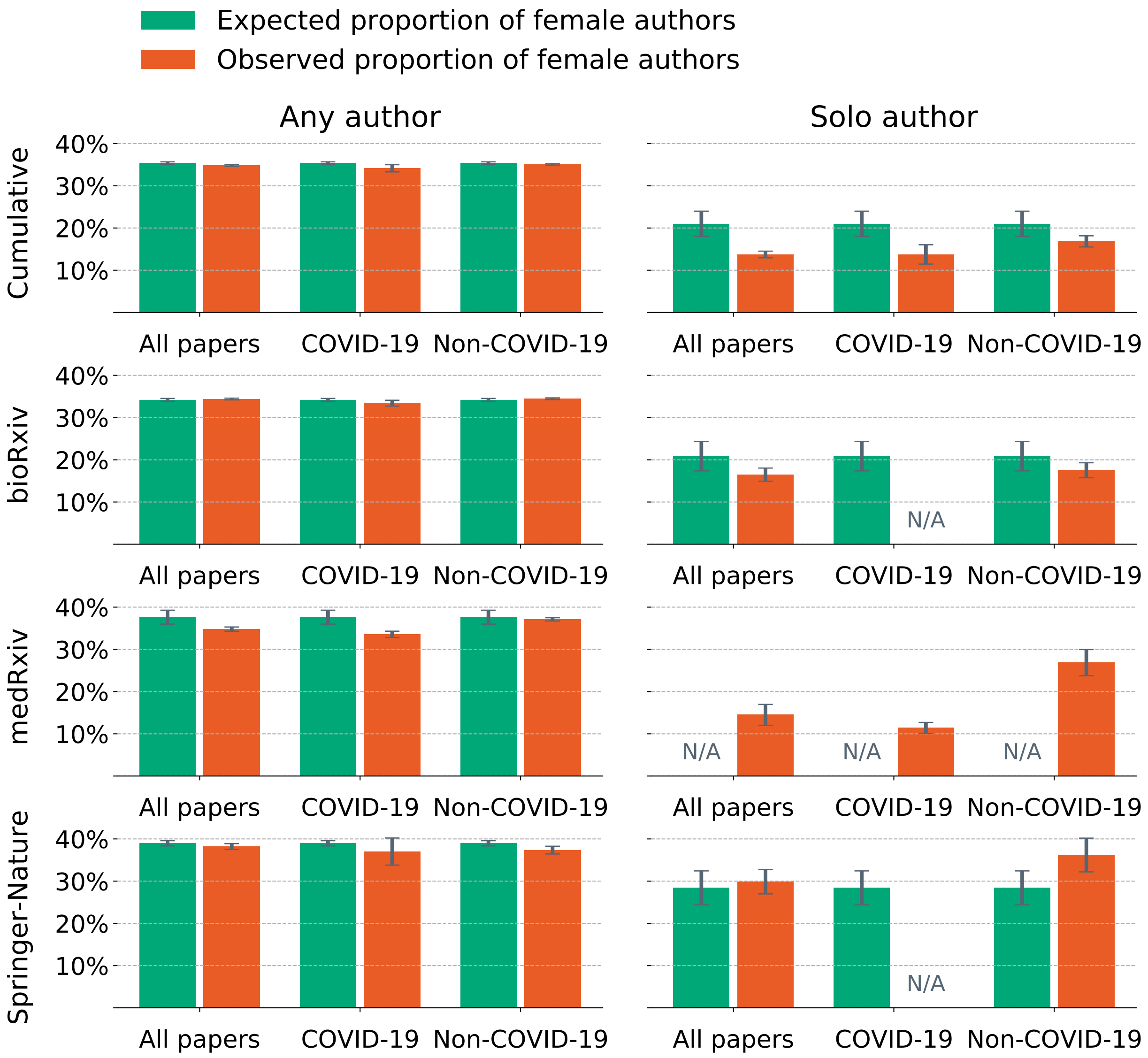

Fig. S2. The comparison of the expected and observed proportion of female authors that publish during the COVID-19 pandemic. Green bars represent the expected proportion of female authors, estimated by the OLS model from the historical data from 2019. Orange bars are the observed proportion of female authors that publish during the COVID-19 pandemic. The papers are divided by the topic in three groups: 1) all papers from the dataset, 2) the papers that deal directly with the COVID-19 and related topics, 3) the papers that are not about COVID-19 or related topics. In the first row are the results from all publishers combined. Other rows represent the results for each publisher separately. Missing bars indicate insufficient number of samples.

Table S6. The optimal cut-off point for RDD design

	W	τ	p	$st.err$
Australia	-13	-0.156	0.345	0.162
Brazil	0	-0.331	**0.007**	0.112
Canada	4	-0.179	**0.013**	0.068
Switzerland	-13	-0.242	0.209	0.188
China	14	-0.110	**0.009**	0.040
Germany	2	-0.162	**0.011**	0.061
Spain	1	-0.247	**0.040**	0.115
France	1	-0.255	**0.000**	0.066
United Kingdom	1	-0.151	**0.001**	0.041
India	2	-0.120	0.103	0.072
Italy	3	-0.264	**0.006**	0.092
Japan	-2	-0.137	0.140	0.089
Netherlands	17	-0.229	0.117	0.143
Sweden	0	-0.240	0.118	0.142
United States	2	-0.120	**0.000**	0.029

W – number of weeks after Jan 31st 2020

Table S7. The trends of the proportion of female authors during the pandemic

	First author		Last author		All authors		Solo author	
	β	p	β	p	β	p	β	p
				All papers				
bioRxiv	-0.001	0.233	0.002	0.0***	0.001	0.015*	0.006	0.023*
medRxiv	0.002	0.053	0.002	0.009**	0.003	0.0***	-0.005	0.087
Springer-Nature	-0.003	0.388	-0.000	0.946	0.003	0.013*	-0.043	0.243
All	-0.002	0.083	0.002	0.002**	0.001	0.0***	0.000	0.999
				COVID-19 papers				
bioRxiv	-0.002	0.449	0.003	0.254	0.003	0.008**	-0.167	–
medRxiv	0.004	0.002**	0.004	0.0***	0.005	0.0***	-0.006	0.058
Springer-Nature	-0.002	0.809	0.019	0.053	0.016	0.003**	–	–
All	0.001	0.278	0.003	0.0***	0.005	0.0***	-0.005	0.119
				Non-COVID-19 papers				
bioRxiv	-0.001	0.301	0.002	0.0***	0.001	0.042*	0.006	0.047*
medRxiv	0.002	0.399	-0.001	0.513	0.000	0.618	-0.009	0.617
Springer-Nature	-0.005	0.157	-0.000	0.861	0.000	0.844	-0.038	0.46
All	-0.002	0.024*	0.001	0.025*	0.000	0.169	0.004	0.132

β - a slope of the linear regression model $f_i = \alpha + \beta t + \epsilon_i$, where t is the time in weeks after mid March 2020
p - p-value
* – $p < 0.05$, ** – $p < 0.01$, *** – $p < 0.001$

Table S8. The expected and observed proportion of female FIRST authors across the countries and disaggregated by the publisher

	All publishers									
	All papers					COVID-19 papers				
	Model		Observation			Model		Observation		
	$\bar{y}_{est}$	$\bar{S}_{est}$	$\bar{y}$	$\sigma_{\bar{x}}$	% diff	$\bar{y}_{est}$	$\bar{S}_{est}$	$\bar{y}$	$\sigma_{\bar{x}}$	% diff
Australia	0.403	0.033	0.381	0.020	-5.412	0.403	0.033	0.365	0.038	-9.47
Brazil	–	–	0.383	0.028	–	–	–	0.3	0.03	–
Canada	0.462	0.03	0.371	0.020	-19.717	0.462	0.03	0.336	0.034	-27.248
China	0.302	0.019	0.302	0.018	-0.088	0.302	0.019	0.271	0.023	-10.364
France	0.466	0.03	0.414	0.017	-11.085	0.466	0.03	0.33	0.024	-29.309
Germany	0.393	0.02	0.360	0.013	-8.595	0.393	0.02	0.252	0.027	-36.01
India	0.365	0.037	0.316	0.024	-13.404	0.365	0.037	0.238	0.025	-34.696
Italy	0.526	0.046	0.395	0.024	-24.931	0.526	0.046	0.391	0.033	-25.583
Japan	0.223	0.028	0.191	0.019	-14.558	0.223	0.028	0.276	0.03	23.735
Netherlands	0.435	0.035	0.448	0.026	3.153	0.435	0.035	0.397	0.034	-8.625
Spain	0.361	0.043	0.367	0.031	1.616	0.361	0.043	0.255	0.032	-29.317
Sweden	0.494	0.046	0.415	0.035	-15.954	0.494	0.046	–	–	–
Switzerland	0.467	0.048	0.430	0.027	-7.864	0.467	0.048	0.402	0.036	-13.972
United Kingdom	0.402	0.02	0.387	0.011	-3.803	0.402	0.02	0.293	0.016	-27.175
United States	0.364	0.011	0.346	0.006	-4.899	0.364	0.011	0.294	0.017	-19.283

	bioRxiv and medRxiv									
	All papers					COVID-19 papers				
	Model		Observation			Model		Observation		
	$\bar{y}_{est}$	$\bar{S}_{est}$	$\bar{y}$	$\sigma_{\bar{x}}$	% diff	$\bar{y}_{est}$	$\bar{S}_{est}$	$\bar{y}$	$\sigma_{\bar{x}}$	% diff
Australia	0.361	0.039	0.357	0.027	-1.185	0.361	0.039	0.354	0.039	-2.122
Brazil	–	–	0.344	0.025	–	–	–	0.292	0.031	–
Canada	0.434	0.037	0.352	0.021	-18.791	0.434	0.037	0.327	0.033	-24.582
China	0.262	0.038	0.290	0.020	10.689	0.262	0.038	0.271	0.024	3.512
France	0.469	0.034	0.417	0.019	-11.216	0.469	0.034	0.329	0.025	-29.943
Germany	0.36	0.023	0.360	0.015	-0.059	0.36	0.023	0.247	0.028	-31.297
India	0.393	0.04	0.313	0.024	-20.396	0.393	0.04	0.239	0.025	-39.249
Italy	0.471	0.048	0.368	0.024	-21.978	0.471	0.048	0.399	0.032	-15.374
Japan	0.229	0.038	0.199	0.021	-13.289	0.229	0.038	0.276	0.03	20.259
Netherlands	0.346	0.047	0.428	0.028	23.725	0.346	0.047	0.405	0.034	16.964
Spain	0.383	0.051	0.355	0.031	-7.185	0.383	0.051	0.256	0.032	-33.112
Sweden	–	–	0.393	0.034	–	–	–	–	–	–
Switzerland	0.463	0.051	0.415	0.027	-10.422	0.463	0.051	0.395	0.038	-14.625
United Kingdom	0.39	0.024	0.385	0.011	-1.146	0.39	0.024	0.289	0.015	-25.96
United States	0.354	0.011	0.342	0.007	-3.617	0.354	0.011	0.275	0.013	-22.324

	Springer-Nature									
	All papers					COVID-19 papers				
	Model		Observation			Model		Observation		
	$\bar{y}_{est}$	$\bar{S}_{est}$	$\bar{y}$	$\sigma_{\bar{x}}$	% diff	$\bar{y}_{est}$	$\bar{S}_{est}$	$\bar{y}$	$\sigma_{\bar{x}}$	% diff
Australia	0.491	0.053	0.465	0.039	-5.238	0.491	0.053	–	–	–
Brazil	–	–	–	–	–	–	–	–	–	–
Canada	0.576	0.05	0.563	0.045	-2.229	0.576	0.05	–	–	–
China	0.343	0.021	0.32	0.02	-6.914	0.343	0.021	0.405	0.055	17.835
France	–	–	–	–	–	–	–	–	–	–
Germany	0.526	0.041	0.39	0.028	-25.724	0.526	0.041	–	–	–
India	–	–	–	–	–	–	–	–	–	–
Italy	0.498	0.045	0.508	0.042	2.043	0.498	0.045	–	–	–
Japan	0.257	0.032	0.319	0.035	24.171	0.257	0.032	–	–	–
Netherlands	–	–	–	–	–	–	–	–	–	–
Spain	–	–	0.472	0.027	–	–	–	–	–	–
Sweden	0.578	0.05	0.507	0.01	-12.381	0.578	0.05	–	–	–
Switzerland	–	–	–	–	–	–	–	–	–	–
United Kingdom	0.487	0.041	0.486	0.03	-0.157	0.487	0.041	–	–	–
United States	0.486	0.03	0.455	0.035	-6.363	0.486	0.03	–	–	–

$\bar{y}_{est}$ is the arithmetic mean of the estimate, and $\bar{y}$ is the arithmetic mean of the observation
$\bar{S}_{est}$ is the mean standard error of the estimate
$\sigma_{\bar{x}}$ is the standard error of the mean (SEM) of the observation

Table S9. The expected and observed proportion of female LAST authors across the countries and disaggregated by the publisher

All publishers

	All papers					COVID-19 papers				
	Model		Observation			Model		Observation		
	$\bar{y}_{est}$	$\bar{S}_{est}$	$\bar{y}$	$\sigma_{\bar{x}}$	% diff	$\bar{y}_{est}$	$\bar{S}_{est}$	$\bar{y}$	$\sigma_{\bar{x}}$	% diff
Australia	0.324	0.03	0.257	0.018	-20.68	0.324	0.03	0.311	0.033	-4.057
Brazil	–	–	0.268	0.025	–	–	–	0.196	0.024	–
Canada	0.312	0.029	0.283	0.014	-9.424	0.312	0.029	0.271	0.036	-13.09
China	0.209	0.014	0.207	0.009	-0.966	0.209	0.014	0.191	0.015	-8.531
France	0.31	0.024	0.291	0.013	-6.103	0.31	0.024	0.242	0.026	-22.11
Germany	0.247	0.021	0.207	0.011	-16.15	0.247	0.021	0.18	0.02	-27.098
India	0.253	0.026	0.220	0.014	-13.239	0.253	0.026	0.225	0.024	-11.154
Italy	0.362	0.042	0.262	0.020	-27.589	0.362	0.042	0.28	0.026	-22.81
Japan	0.129	0.018	0.081	0.007	-37.369	0.129	0.018	–	–	–
Netherlands	0.273	0.031	0.269	0.024	-1.337	0.273	0.031	0.38	0.037	39.323
Spain	0.301	0.036	0.237	0.020	-21.426	0.301	0.036	0.24	0.024	-20.388
Sweden	0.338	0.04	0.325	0.026	-3.958	0.338	0.04	0.39	0.036	15.172
Switzerland	0.256	0.038	0.203	0.016	-20.816	0.256	0.038	0.322	0.034	25.712
United Kingdom	0.284	0.016	0.246	0.007	-13.383	0.284	0.016	0.233	0.016	-18.131
United States	0.245	0.009	0.240	0.006	-2.014	0.245	0.009	0.219	0.011	-10.394

bioRxiv and medRxiv

	All papers					COVID-19 papers				
	Model		Observation			Model		Observation		
	$\bar{y}_{est}$	$\bar{S}_{est}$	$\bar{y}$	$\sigma_{\bar{x}}$	% diff	$\bar{y}_{est}$	$\bar{S}_{est}$	$\bar{y}$	$\sigma_{\bar{x}}$	% diff
Australia	0.271	0.029	0.233	0.017	-14.054	0.271	0.029	0.313	0.032	15.653
Brazil	–	–	0.246	0.019	–	–	–	0.196	0.024	–
Canada	–	–	0.270	0.015	–	–	–	0.277	0.036	–
China	0.209	0.026	0.207	0.011	-0.81	0.209	0.026	0.195	0.016	-6.828
France	0.319	0.027	0.289	0.014	-9.352	0.319	0.027	0.237	0.026	-25.55
Germany	0.23	0.025	0.204	0.012	-11.466	0.23	0.025	0.184	0.022	-20.226
India	0.241	0.028	0.219	0.015	-9.467	0.241	0.028	0.223	0.024	-7.6
Italy	0.351	0.048	0.252	0.021	-28.28	0.351	0.048	0.276	0.026	-21.392
Japan	–	–	0.085	0.008	–	–	–	–	–	–
Netherlands	–	–	0.261	0.024	–	–	–	0.376	0.036	–
Spain	0.261	0.031	0.233	0.018	-10.741	0.261	0.031	0.234	0.021	-10.346
Sweden	–	–	0.294	0.027	–	–	–	0.417	0.035	–
Switzerland	–	–	0.202	0.017	–	–	–	0.333	0.035	–
United Kingdom	0.259	0.019	0.240	0.007	-7.423	0.259	0.019	0.23	0.014	-11.247
United States	0.239	0.01	0.237	0.006	-0.658	0.239	0.01	0.218	0.011	-8.53

Springer-Nature

	All papers					COVID-19 papers				
	Model		Observation			Model		Observation		
	$\bar{y}_{est}$	$\bar{S}_{est}$	$\bar{y}$	$\sigma_{\bar{x}}$	% diff	$\bar{y}_{est}$	$\bar{S}_{est}$	$\bar{y}$	$\sigma_{\bar{x}}$	% diff
Australia	0.456	0.047	0.427	0.031	-6.394	0.456	0.047	–	–	–
Brazil	–	–	–	–	–	–	–	–	–	–
Canada	0.473	0.044	0.439	0.039	-7.262	0.473	0.044	–	–	–
China	0.219	0.014	0.212	0.015	-3.314	0.219	0.014	–	–	–
France	–	–	–	–	–	–	–	–	–	–
Germany	–	–	0.262	0.018	–	–	–	–	–	–
India	–	–	–	–	–	–	–	–	–	–
Italy	0.529	0.047	0.334	0.025	-36.754	0.529	0.047	–	–	–
Japan	0.134	0.019	0.211	0.031	57.366	0.134	0.019	–	–	–
Netherlands	–	–	0.355	0.037	–	–	–	–	–	–
Spain	–	–	–	–	–	–	–	–	–	–
Sweden	0.581	0.049	0.53	0.032	-8.862	0.581	0.049	–	–	–
Switzerland	–	–	–	–	–	–	–	–	–	–
United Kingdom	0.419	0.038	0.365	0.035	-12.856	0.419	0.038	–	–	–
United States	0.349	0.029	0.349	0.021	-0.107	0.349	0.029	–	–	–

$\bar{y}_{est}$ is the arithmetic mean of the estimate, and $\bar{y}$ is the arithmetic mean of the observation
$\bar{S}_{est}$ is the mean standard error of the estimate
$\sigma_{\bar{x}}$ is the standard error of the mean (SEM) of the observation

Table S10. The expected and observed proportion of female authors REGARDLESS Of THE ORDER across the countries and disaggregated by the publisher

	All publishers									
	All papers					COVID-19 papers				
	Model		Observation			Model		Observation		
	$\bar{y}_{est}$	$\bar{S}_{est}$	$\bar{y}$	$\sigma_{\bar{x}}$	% diff	$\bar{y}_{est}$	$\bar{S}_{est}$	$\bar{y}$	$\sigma_{\bar{x}}$	% diff
Australia	0.383	0.018	0.367	0.006	-4.360	0.383	0.018	0.371	0.024	-3.127
Brazil	0.457	0.022	0.399	0.015	-12.852	0.457	0.022	0.326	0.024	-28.644
Canada	0.378	0.014	0.354	0.007	-6.438	0.378	0.014	0.318	0.018	-15.782
China	0.270	0.009	0.278	0.004	2.657	0.270	0.009	0.277	0.009	2.483
France	0.410	0.012	0.404	0.005	-1.479	0.410	0.012	0.367	0.021	-10.441
Germany	0.347	0.009	0.331	0.005	-4.495	0.347	0.009	0.301	0.012	-13.117
India	0.310	0.017	0.306	0.010	-1.425	0.310	0.017	0.303	0.019	-2.287
Italy	0.440	0.021	0.414	0.009	-5.786	0.440	0.021	0.382	0.024	-13.093
Japan	0.155	0.010	0.177	0.006	13.697	0.155	0.010	0.165	0.016	6.079
Netherlands	0.373	0.014	0.376	0.010	0.876	0.373	0.014	0.339	0.025	-9.127
Spain	0.411	0.015	0.413	0.010	0.545	0.411	0.015	0.394	0.023	-4.120
Sweden	0.402	0.021	0.360	0.011	-10.394	0.402	0.021	0.321	0.020	-20.059
Switzerland	0.351	0.020	0.360	0.011	2.425	0.351	0.020	0.330	0.025	-6.078
United Kingdom	0.384	0.008	0.362	0.004	-5.629	0.384	0.008	0.351	0.012	-8.695
United States	0.350	0.006	0.349	0.004	-0.194	0.350	0.006	0.350	0.012	0.064
	bioRxiv and medRxiv									
	All papers					COVID-19 papers				
	Model		Observation			Model		Observation		
	$\bar{y}_{est}$	$\bar{S}_{est}$	$\bar{y}$	$\sigma_{\bar{x}}$	% diff	$\bar{y}_{est}$	$\bar{S}_{est}$	$\bar{y}$	$\sigma_{\bar{x}}$	% diff
Australia	0.357	0.019	0.351	0.009	-1.701	0.357	0.019	0.359	0.025	0.733
Brazil	0.417	0.026	0.385	0.015	-7.662	0.417	0.026	0.324	0.024	-22.330
Canada	0.350	0.017	0.342	0.008	-2.131	0.350	0.017	0.314	0.019	-10.086
China	0.263	0.012	0.281	0.006	6.663	0.263	0.012	0.282	0.010	6.983
France	0.414	0.014	0.404	0.005	-2.505	0.414	0.014	0.368	0.021	-11.164
Germany	0.338	0.011	0.331	0.005	-1.812	0.338	0.011	0.299	0.012	-11.472
India	0.328	0.018	0.305	0.010	-6.935	0.328	0.018	0.303	0.019	-7.499
Italy	0.419	0.028	0.407	0.011	-2.804	0.419	0.028	0.375	0.026	-10.529
Japan	0.171	0.016	0.182	0.008	6.554	0.171	0.016	0.164	0.017	-4.245
Netherlands	0.342	0.016	0.370	0.009	7.929	0.342	0.016	0.341	0.025	-0.378
Spain	0.405	0.019	0.410	0.011	1.246	0.405	0.019	0.392	0.024	-3.370
Sweden	0.356	0.026	0.347	0.012	-2.674	0.356	0.026	0.319	0.021	-10.404
Switzerland	0.334	0.021	0.355	0.011	6.241	0.334	0.021	0.323	0.024	-3.264
United Kingdom	0.366	0.010	0.360	0.004	-1.787	0.366	0.010	0.346	0.012	-5.625
United States	0.341	0.005	0.344	0.003	1.030	0.341	0.005	0.345	0.009	1.059
	Springer-Nature									
	All papers					COVID-19 papers				
	Model		Observation			Model		Observation		
	$\bar{y}_{est}$	$\bar{S}_{est}$	$\bar{y}$	$\sigma_{\bar{x}}$	% diff	$\bar{y}_{est}$	$\bar{S}_{est}$	$\bar{y}$	$\sigma_{\bar{x}}$	% diff
Australia	0.469	0.03	0.492	0.033	4.921	0.469	0.03	–	–	–
Brazil	0.528	0.036	0.523	0.041	-0.917	0.528	0.036	–	–	–
Canada	0.504	0.024	0.541	0.034	7.477	0.504	0.024	–	–	–
China	0.28	0.01	0.241	0.012	-13.679	0.28	0.01	0.215	0.027	-23.235
France	0.405	0.025	0.349	0.041	-14.045	0.405	0.025	–	–	–
Germany	0.381	0.025	0.356	0.022	-6.539	0.381	0.025	0.371	0.036	-2.705
India	0.26	0.042	0.492	0.033	89.181	0.26	0.042	–	–	–
Italy	0.483	0.028	0.415	0.026	-14.185	0.483	0.028	–	–	–
Japan	0.132	0.017	0.165	0.024	24.88	0.132	0.017	–	–	–
Netherlands	0.477	0.034	0.471	0.029	-1.239	0.477	0.034	–	–	–
Spain	0.458	0.041	0.435	0.024	-5.142	0.458	0.041	–	–	–
Sweden	0.496	0.04	0.480	0.027	-3.129	0.496	0.04	–	–	–
Switzerland	0.421	0.042	0.449	0.031	6.429	0.421	0.042	–	–	–
United Kingdom	0.473	0.02	0.414	0.025	-12.48	0.473	0.02	0.416	0.024	-12.061
United States	0.434	0.014	0.442	0.028	1.831	0.434	0.014	0.37	0.042	-14.663

$\bar{y}_{est}$ is the arithmetic mean of the estimate, and $\bar{y}$ is the arithmetic mean of the observation
$\bar{S}_{est}$ is the mean standard error of the estimate
$\sigma_{\bar{x}}$ is the standard error of the mean (SEM) of the observation

Table S11. The list of countries with the largest number of authors and papers in the dataset

country code	Name	# authors	# papers
US	United States	111212	34880
GB	United Kingdom	33386	12113
CN	China	22381	10439
DE	Germany	21919	7816
FR	France	16846	5715
CA	Canada	13037	5226
JP	Japan	12413	3752
AU	Australia	10603	4224
IT	Italy	8961	2862
NL	Netherlands	8877	3520
ES	Spain	8063	2809
IN	India	7598	3440
CH	Switzerland	6632	2863
BR	Brazil	6582	1779
SE	Sweden	5224	2226
RU	Russian Federation	3920	2066
DK	Denmark	3478	1464
NO	Norway	3019	1254
KR	Korea	2997	1280
BE	Belgium	2977	1210
IR	Iran, Islamic Republic Of	2957	913
AT	Austria	2695	1116
IL	Israel	2485	1053
PL	Poland	2136	839
FI	Finland	2113	883
ZA	South Africa	1923	1017
MX	Mexico	1853	742
SG	Singapore	1665	756
PT	Portugal	1516	631
GR	Greece	1453	650
